# Supplementary material for: Economic assessment of potential changes to essential medicines for diabetes in Uganda
Source: PLoS One. 2025 Jun 25;20(6):e0326806. doi: 10.1371/journal.pone.0326806 (PMC12193067; doi:10.1371/journal.pone.0326806)
Supplement: S1 Table — Checklist used to guide and assess the completeness of the budget impact analysis based on ISPOR guidelines. (DOCX) [file pone.0326806.s001.docx]

**S1 Table. Source countries from which oral hypoglycemic medicines are imported into Uganda (2021-2023)**

| **Generic Name** | **Source Countries** |
| --- | --- |
| Metformin 500 mg | DE, IN, FR, NL, CY, SI, BE, KE, US, ZA, GB |
| Glibenclamide 5 mg | BE, CN, EG, FR, IN, NL, US |
| Gliclazide 80 mg | IN, GB |
| Glimepiride 2 mg | DE, GB, IN, JO, PK, SI |
| Pioglitazone 30 mg | IN, GB |
| Dapagliflozin 5 mg | IN, KB |
| Vildagliptin 50 mg | SI, CH, DE, IN |

**Notes:** BE=Belgium, CH=Switzerland, CN=China, CY=Cyprus, DE=Denmark, EG=Egypt, FR=France, GB=United Kingdom, IN=India, JO=Jordan, KE=Kenya, NL=The Netherlands, PK=Pakistan, SI=Slovenia, US=United States, ZA=South Africa
