# Supplementary material for: Economic assessment of potential changes to essential medicines for diabetes in Uganda
Source: PLoS One. 2025 Jun 25;20(6):e0326806. doi: 10.1371/journal.pone.0326806 (PMC12193067; doi:10.1371/journal.pone.0326806)
Supplement: S2 Table — Checklist used to guide and assess the completeness of the budget impact analysis based on ISPOR guidelines. (DOCX) [file pone.0326806.s002.docx]

**S2. Budget Impact Analysis Reporting Checklist based on ISPOR Guidelines**

| **Checklist Item** | **Response / Assessment** | **Page Number (s)** |
| --- | --- | --- |
| Budget Impact Analysis Objective | To evaluate the potential cost savings and economic impact of substituting Oral Hypoglycemic Agents (OHAs) on the Essential Medicines and Health Supplies List (EMHSLU) in Uganda | 9 |
| Epidemiology and Management of Health Problem | Diabetes is a high-burden disease in Uganda with a prevalence of 4.6% in 2021 and an estimated 48% of patients remaining undiagnosed | 27 |
| Clinical Impact | The efficacy and safety profiles of the OHAs under consideration are summarized in Table 7. | 22 |
| Economic Impact | No previous Budget Impact Analyses specific to diabetes management or OHAs have been conducted in Uganda. | - |
| Patient Population | The eligible population for this analysis consists of diabetic patients in Uganda receiving care in various levels of the care, including Health Center II, Health Center III, Health Center IV, General Hospitals, Regional Referral Hospitals, and National Referral Hospital. (see **Table 3**) | 9, 14 |
| Intervention Mix | The current intervention mix includes OHAs listed in EMHSLU 2016 and 2023 and their substitutions. (see **S3 Table)** | 14 |
| Time Horizon | The time horizon for this Budget Impact Analysis is set at one year (1/1/2023 – 31/12/2023). This timeline was selected based on the availability of data on the number of patients who received care during 2023 and the medicines' unit prices for the same year. A one-year horizon also aligns with the government’s annual budgeting process. | 9 - 10 |
| Perspective | This Budget Impact Analysis is conducted from the government’s perspective in Uganda for managing diabetes with OHAs in public sector. | 9 |
| Analytic Framework Description | The structure involves calculating the total costs of OHAs for diabetic patients in public sector in Uganda. The model incorporates population estimates, drug unit prices, and treatment costs based on the WHO Defined Daily Dose (DDD) methodology. Two scenarios are evaluated: substitutions within the current EMHSLU and substitutions involving medicines outside the EMHSLU but available in Uganda. (see **S3 Table**) | Supplement |
| Input Data | *Patient Population Estimates*: Derived from routine health facility data for 2023, capturing the number of diabetic patients across different levels of healthcare facilities (e.g., HC II, HC III, HC IV, hospitals).  *Medication Unit Prices*: Sourced from the 2024 national warehouse catalog to reflect the most recent procurement costs of oral hypoglycemic agents (OHAs).  *WHO Defined Daily Dose (DDD)*: Used as a standard reference for calculating the daily dosage requirements of each medication.  *Exchange Rate*: Data from the World Bank (2023) was used to convert all costs from Ugandan Shillings (UGX) to USD for international comparability. These inputs are clearly outlined in the manuscript, and sufficient detail is provided to allow replication of all calculations in the model. (see **S3 Table and S3 Table**) | Table 3 (Page 14),  S3 Table (Supplement) |
| Data Source | *Routine Health Facility Reports (2023)*: Used to estimate the eligible population of diabetic patients requiring OHAs at various levels of care.  *National Warehouse Catalog (2023)*: Provided unit prices for medications listed in the analysis.  *WHO DDD Database*: Referenced for standardized daily dose calculations.  *World Bank*: Supplied the exchange rate for currency conversions. | 9 - 10 |
| Data Collection | This analysis relied exclusively on secondary data sources. | - |
| Analysis | The Annual Treatment Cost per Patient was determined using the formula:  $Annual Treatment Cost per Patient \left( UGX \right)=\frac{WHO DDD}{tablet strenght}\times365\times Unit Price (UGX)$  The Total Cost per Medicine was then calculated by multiplying the annual cost per patient by the estimated number of patients receiving the medication. Two scenarios were analyzed:  Scenario 1: Substitutions within the EMHSLU.  Scenario 2: Substitutions involving medicines not currently included in the EMHSLU but available in Uganda. (see **S3 Table**) | 9 - 10 |
| Uncertainty | Uncertainty in this Budget Impact Analysis was addressed through scenario-based analysis, which reflects alternative policy decisions and their potential cost implications. | - |
| Results | **Scenario 1: Substitutions Within EMHSLU**  *Sulfonylureas*: Replacing Glimepiride 2 mg with Glibenclamide 5 mg at HC3 and higher levels could save USD 2 million. Replacing Gliclazide 80 mg with Glibenclamide 5 mg at RR and NR could save USD 650,000, or USD 400,000 if replaced with Glimepiride 2 mg.  *Other Drug Classes*: No cost-saving substitutions identified for Biguanides, Thiazolidinediones, SGLT2 inhibitors, or DPP-4 inhibitors.  **Scenario 2: Substitutions Outside EMHSLU**  *Sulfonylureas*: Replacing Glimepiride 2 mg with Glimepiride 4 mg could save USD 1.5 million. Replacing Gliclazide 80 mg with Glimepiride 4 mg at RR and NR could save USD 600,000.  *SGLT2 Inhibitors*: Replacing Dapagliflozin 5 mg with Dapagliflozin 10 mg at RR and NR could save USD 230,000.  *Other Drug Classes*: No alternatives identified for Biguanides, Thiazolidinediones, or DPP-4 inhibitors. (See **Table 5**) | 18 |
| Conclusions and Limitations | **Conclusion:** This Budget Impact Analysis highlights significant cost-savings opportunities for Uganda’s public healthcare system through strategic substitutions of OHAs in the EMHSLU.  **Limitation:** The unit prices used in the BIA were sourced from 2023; however, changes in drug prices due to inflation could significantly impact the estimated cost-savings. | 26 - 27 |
| Figure of the Analytical Framework | See **S3 Table** and **Table 5** | Supplement |
| Table of Assumptions | See **S3 Table** and **Table 5** | Table 3 (Page 14),  S3 Table (Supplement) |
| Tables of Inputs | See **S3 Table**, **Table 2**, **Table 3**, and **Table 5** | Table 2 (Page 13), Table 3 (Page 14), Table 5 (Page 20)  S3 Table (Supplement) |
| Schematic Representation of Uncertainty Analyses | While the analysis incorporates scenario-based uncertainty (e.g., evaluating substitutions within and outside the EMHSLU), parameter-specific sensitivity testing was not conducted due to the high reliability of input data, such as medication prices and patient population estimates which were sourced from the Ministry of Health in Uganda. | - |
| Appendices and References | See **S3 Table**, **Table 2**, **Table 3**, and **Table 5** | Table 2 (Page 13), Table 3 (Page 14), Table 5 (Page 20)  S3 Table (Supplement) |
| Reporting BIAs Alongside CEAs | See **Table 6** | 20 |
