# Supplementary material for: Economic assessment of potential changes to essential medicines for diabetes in Uganda
Source: PLoS One. 2025 Jun 25;20(6):e0326806. doi: 10.1371/journal.pone.0326806 (PMC12193067; doi:10.1371/journal.pone.0326806)
Supplement: Attachment — Submitted filename: Response to Reviewers.docx [file pone.0326806.s005.docx]

Manuscript ID: PONE-D-25-01852
Title: *Economic Assessment of Potential Changes to Essential Medicines for Diabetes in Uganda*
Corresponding Author: Atousa Bonyani

We thank the Academic Editor and both reviewers for their thorough and constructive feedback on our manuscript. We appreciate the thoughtful insights, which helped us improve the clarity, accuracy, and overall contribution of our work. We carefully address each comment below, made the corresponding revisions to the manuscript in track changes, and added new references to support new content added. Moreover, we made few additional edits in track changes to improve the draft.

**Academic Editor Comments**

**Comment 1:** Page 15, line 282 – What does “health center 4” mean? “Similarly, glibenclamide 5 mg was accessible in more than 75% of the health center 4 and hospitals that were assessed.” (Minor)

**Response:** Thank you for pointing this out. As shown in Table 3, Health Center 4 (HC4) is part of Uganda’s secondary level of care. To make the term clearer for readers, we’ve revised the sentence and replaced “health center 4” with *“HC4”*.

**Comment 2:** In the discussion of sulfonylureas on page 17, the difference in cost is discussed, but the clinical differences are not incorporated, leaving the assessment incomplete. For example, the major advantage of gliclazide is the enhanced safety among patients with renal insufficiency and decreased risk of hypoglycemia (as described in Table 7). From a health economics perspective, the number of patients not experiencing severe hypoglycemia should be factored into the comparative assessment instead of treating these medications as cost-equivalent. (Moderate)

**Response:** Thank you for highlighting this important consideration. We have added the following to our limitations with respect to the BIA: “*Lastly, this analysis did not account for the potential downstream costs associated with adverse clinical outcomes, such as hypoglycemia, which may be more common with certain medicines like glibenclamide and could impact some of the projected cost savings.*”

**Comment 3:** Why aren't biguanides or SGLT2 inhibitors included in Table 7? (Minor)

**Response:** Thank you for your comment. Table 7 is focused on sulfonylureas and DPP-4 inhibitors because these classes include multiple alternative medicines currently available in Uganda, allowing for within-class comparisons. In contrast, only one medicine from the biguanide (metformin) and SGLT2 inhibitor (dapagliflozin) classes is presently registered in the country. Since no alternative medicines within those classes are in use or under consideration for the Essential Medicines List, we excluded them from the comparative safety and efficacy summary in Table 7. We have added the following for clarity: *“In contrast, since only one medicine from each of the biguanides, thiazolidinediones, and SGLT2 inhibitors classes is available in the country, a meaningful within-class comparison for these categories is not possible.”*

**Comment 4:** Is there a reason GLP-1s are mentioned anywhere? (Minor)

**Response:** Thank you for your comment. Our analysis focused exclusively on oral hypoglycemic agents that are currently available in Uganda and may be considered for inclusion in the Essential Medicines and Health Supplies List. We did not include injectable agents such as GLP-1 receptor agonists or insulin formulations, as these were outside the scope of our evaluation. Similarly, the new oral formulation of GLP-1s (Rybelsus) is not available in Uganda.

**Reviewer #1 Comments**

**2. Major Areas of Improvement**

**2.1. Methodology**

**Comment 1 & 2:** On page 9 and in Table 3, the paper calculates the size of the population receiving each medicine based on “routine health facility data and the medicines that are required to be available at the respective level of care.” Please provide more detailed information on the calculations and assumptions that you used to arrive at the numbers in Table 3. If you used point-of-care dispensation data at point-of-care clinics, I’m concerned that your numbers likely to be right-censored (due to products being out of stock, etc.) Hence, your estimates would be significantly underestimating the total number of patients with diabetes in the country. (Moderate-I would like to see some comment on how you address the underestimation of patients as a result of stockouts)

For instance, the data presented in Table 3 suggests that the total population of adult diabetic patients in Uganda is approximately 320,000 across all medications and levels of care. However, based on the 4.6% prevalence of diabetes in Uganda, as noted on page 27, and a total population of around 50 million people, the estimated number of diabetic patients should be closer to 2,300,000. This discrepancy indicates that your calculations may underestimate the actual patient population. Please clarify this inconsistency, as it raises concerns about the accuracy and reliability of the findings. (Moderate-I would include a comment on this discrepancy in the paper while still acknowledging that countries like Uganda have the highest rate of underdiagnosed diabetes and struggle with inconsistent follow-up)

**Response:** We acknowledge the reviewer’s concern. The details of calculation of the population is mentioned in the method section and under Affordability part: “*To calculate the size of the population receiving each medicine, we reviewed the number of diabetic patients in each level of care in 2023 based on routine health facility data and the medicines that are required to be available at the respective level of care. For each medication, we summed up the number of patients across all levels of care that are required to provide that medication. This information provides us with the total population that may potentially receive the drug. We then multiplied this total number by the estimated proportion of patients receiving each drug class to determine the target population for the BIA*”. As you rightly pointed out, there is high rate of undiagnosed patients in the country that we also mentioned in our conclusion. In addition, many people diagnosed with T2DM prefer going to the private sector due to less stock-outs in the private sector. “*With a prevalence of 4.6% in 2021 and an estimated 48% of patients remaining undiagnosed, diabetes is considered a high-burden disease in Uganda.*” “*Despite the costs, many patients diagnosed with T2DM prefer private-for-profit clinics over public healthcare facilities because these clinics often have better availability of medicines.*” Our number of 320,000 diabetic patients in the analysis refers to those diagnosed patients who are receiving care in public sector only.

In terms of the stock-outs, our data is not coming from the point-of-care dispensing data. In fact, we have calculated those patients who seek care at public sector. We have referred to the accessibility challenges in the discussion and under accessibility section “*As the new EMHSLU was revised in 2023, there are no published surveys yet on the accessibility of the newly added medicines. However, our results suggest that budget limitations have significantly impacted the accessibility of medicines, such as Dapagliflozin 5 mg and Vildagliptin 50 mg. Since, supply chain system plays a crucial role in ensuring the geographical accessibility of medicines, effective supply chain management, including accurate forecasting, procurement of necessary dosage forms, timely delivery to health facilities, and appropriate storage, needs to be strengthened in Uganda for improved functionality.*”

We have added *“Additionally, Frequent stock-outs of essential medicines in public health facilities often forces patients to turn to private clinics and pharmacies to continue their treatment which can compromise affordability and continuity of care.”* In the discussion and under Accessibility section to acknowledge that less-frequent stock-outs would support the care received by patients and supposedly, more patients could receive care at public sector instead of the need to go to the private sector. We have added a supporting reference to clarify this point (now ref #74).

**Comment 3:** Similarly, the percentage last-mile availability of health products based on “routine health facility reporting” data—reported as 62% and 76%—are likely to be biased. For example, the 62% last-mile availability of Metformin 500 mg could be due to a variety of different factors such as high popularity among the population leading to stock-outs, low popularity leading health facilities to not prioritize the product, or lack of sufficient procurement quantities at the national level. With that in mind, an alternative to completely replacing a drug on the EMHSL could involve improving last-mile availability or increasing procurement quantities. How do these alternative interventions compare in your context, and what impact might they have on accessibility, affordability, and health outcomes? (Moderate)

**Response:** Thank you for this valid comment. The primary focus of our analysis was to evaluate the economic implications of potential substitutions within the Essential Medicines and Health Supplies List (EMHSL). We did not assess how such substitutions might influence medicine demand and, in turn, affect availability or accessibility at health facilities. We recognize that changes in the EMHSL could alter prescribing patterns and procurement volumes, which may impact the supply chain dynamics. To clarify this limitation, we have added the following sentence to the limitations section: *“This study did not assess how changes in demand resulting from substitutions in the EMHSL might influence medicine availability or accessibility in Uganda.”*

**Comment 4:** Please provide detailed information on where your estimated cost savings originate from. Are these purely based on the difference between the average cost of procuring the original health products and your recommended substitutes? If so, there are a few additional factors that may need to be taken into account during the cost calculation process. For example, how comparable are the inventory holding costs for the two types of products given potential differences in packaging size (e.g., Glimepiride 4mg vs. Gliclazide 80 mg)? How about obsolescence and expiration related costs? (Major-more description of these estimations are important to help the reader understand your calculations)

**Response:** We appreciate this important point. The estimated cost savings presented in the Budget Impact Analysis are based on the difference in annual procurement costs between the medicines currently on the EMHSL and the proposed alternatives, assuming similar treatment coverage. The analysis uses average unit procurement prices and estimated patient volumes from routine health facility data and national supply lists.

We acknowledge that our calculations do not account for additional cost components such as inventory holding costs, packaging-related storage differences, or potential wastage due to expiration. These factors could influence the true budget impact, particularly when products vary in packaging volume, shelf life, or supply chain complexity. We have added this clarification to the limitations section to acknowledge that our estimates reflect procurement-level cost differences but do not capture all downstream supply chain costs. *“This BIA is focused on procurement-level costs and did not account for additional supply chain considerations such as inventory holding costs, packaging-related storage differences, etc.”*

**Comment 5:** In Table 6, the paper utilizes data from countries other than Uganda in order to evaluate the cost-effectiveness of various drugs in the Ugandan context. Some of the countries listed, however, exhibit very different health infrastructure, costs, and health insurance models than Uganda (e.g., Canada, US, UK). Please clarify if and why these are valid benchmarks for Uganda. (Minor-please comment on the inherent limitations with the lack of data from countries more similar to Uganda)

**Response:** Thank you for your comment. As part of our analysis, we mentioned the following to acknowledge concerns for translating ICERs between different settings: “*As an example, for the study in Ecuador, we adjusted the pharmaceutical costs based on the unit prices in Uganda, and we deflated the prices to reach an estimation in 2021. Then, we adjusted other medical costs based on health expenditure per capita. The effectiveness in the original paper was mentioned as 0.02. Therefore, based on the new calculations, the ICER is $6,132 per QALY, while when adjusting all costs based on health expenditure per capita, the ICER was $2,324 per QALY. This significant difference underscores the challenge of geographically adopting an ICER due to the substantial variation in costs and utilization across different settings and highlights that further investigation is needed to accurately assess the cost-effectiveness of OHAs in Uganda*.”

In addition, we had mentioned this limitation in our limitations: “*we translated ICERs from other settings into an estimated ICER for Uganda, using the simplifying assumption that there is a link between ICERs and health expenditure per capita, whereas a detailed analysis for cost and utility adjustment is required to precisely adapt the ICERs geographically*.”

**Comment 6:** Table 7 provides a summary of the clinical profiles (safety and efficacy) across different classes and types of products. This is a good starting point, but I believe for the results of the study to be valid, this analysis needs to be conducted more rigorously. For example, would it be possible to develop more precise measurements of safety and efficacy beyond the current categorical classification of low, moderate, and high? Further, please clarify whether or not the safety and efficacy metrics in Table 7 are universal clinical profiles that are likely to hold in various settings. Or do these need to be modified depending on the specific characteristics of each country (e.g., based on demographic factors, disease prevalence, comorbidities, etc.)? (Major)

**Response:** We appreciate this thoughtful comment. The clinical profiles in Table 7 are based on comparative evidence from published systematic reviews and meta-analyses and are intended to provide a general summary of safety and efficacy across drug classes, based on universal metrics. As outlined in the Methods section, “*The safety and efficacy of available medicines within each OHA drug class in Uganda were appraised in accordance with published systematic reviews and meta-analyses covered in our literature review*.” To improve clarity, we have revised the Methods section to further specify the criteria used by adding the following: *“We focused on universally reported safety and efficacy indicators, including risk of hypoglycemia, cardiovascular events, and all-cause mortality for safety; and HbA1c reduction for efficacy.”* These parameters were selected due to their consistency across comparative trials and their relevance to both global and local decision-making contexts.

We followed the classification approach used in several of the systematic reviews and meta-analyses we relied upon, many of which summarized comparative outcomes using categorical terms such as “low,” “moderate,” or “high” for risk and efficacy. This approach allowed for a consistent and interpretable comparison across medicines and drug classes. We also added the following to the limitations to address this comment: *“This study also relied on summarized clinical profiles reported in existing systematic reviews and meta-analyses. A more rigorous review of reviews may be needed to capture the full range of safety and efficacy outcomes for oral hypoglycemic agents and produce more granular estimates tailored to the Ugandan context.”*

**2.2. Terminologies**

**Comment 1:** The terminologies used in the paper need to be defined more clearly, more upfront, and used consistently throughout the manuscript. For example, “availability” is defined at the national level, where as “accessibility” is also referring to availability but at the more granular and last-mile health facility level. This is somewhat confusing. Should these be referred to as national availability and last-mile availability instead? (Moderate)

**Response:** Thank you for this thoughtful comment. We agree that some of the terms used in the manuscript may seem overlapping or potentially confusing. However, our terminology was based on the WHO guideline for revising national essential medicines lists, which outlines five core criteria: availability, accessibility, affordability, cost-effectiveness, and clinical profiles of medicines. To remain consistent with that framework, we retained these terms and applied them accordingly throughout our analysis. We have added *“and at the health facility levels”* to the accessibility definition in the method section to make these terms clearer for the readers. In addition, we have revised the relevant paragraphs to ensure consistent use of the term “*accessibility*” when referring to access to medicines at the health facility levels.

**Comment 2 & 3:** Similarly, the definition of “affordability” in the paper is rather unorthodox, being defined as the affordability of a health product with respect to government. However, affordability is most commonly defined—in both academia and practice—as products being affordable to patients and clients who are end-users (and not government). In this context, cost-effectiveness seems like a more appropriate terminology. (Minor-I understand that medications are provided for free in the public sector making defining this term slightly problematic)

Quite confusingly, the paper has a separate category named “cost-effectiveness” which is defined as “the costs and health outcomes associated with pharmaceutical interventions, comparing the relative expenses and benefits of different medications to determine.” How is this differentiated from “affordability”? (Major)

**Response:** We are grateful for this insightful comment. As our analysis is guided by the WHO framework for revising national essential medicines lists, we have incorporated both affordability and cost-effectiveness as distinct criteria. While cost-effectiveness refers to an evaluation of value for money, affordability refers to the ability to pay for a product or service. Given that medicines in the public sector are paid for by the government, we assessed affordability using a Budget Impact Analysis (BIA), which aligns with the healthcare system perspective outlined in our Methods section “**Affordability** is defined through different dimensions: the product value dimension considers cost-effectiveness and Quality Adjusted Life Year (QALY), the healthcare system perspective dimension focuses on optimizing the health system budget using budget impact analysis (BIA), and the family perspective dimension considers minimum worker wages or Gross Domestic Product (GDP).”

**Comment 4:** On page 7, the paper defines “affordability” in three different ways: ”the product value dimension considers cost- effectiveness and Quality Adjusted Life Year (QALY), the healthcare system perspective dimension focuses on optimizing the health system budget using budget impact analysis (BIA), and the family perspective dimension considers minimum worker wages or Gross Domestic Product (GDP).“ These are very different definitions with substantially different implications. Which of these three is the definition adopted by the paper? It seems like it’s government affordability. Again, there are a lot of inconsistencies in definitions that are confusing to the reader.

**Response:** Thank you for pointing out this important issue. Our approach was to show that for each criterion, there are multiple approaches to measurement, as identified through our literature review. These are also summarized in Table 1, which outlines different metrics used to assess availability, accessibility, and affordability. In the subsequent section (Characterize EMHSLU), we have specified which metric was applied for each criterion based on data availability and contextual relevance. In the case of affordability, we used Budget Impact Analysis (BIA) to evaluate affordability from the perspective of the Ugandan government, as the payer for medicines at public sector facilities. To make this approach clearer to the reader, we have revised the introductory paragraph of the Methods section as follows:

Original: “Taking WHO recommendations into consideration, we employed a literature review followed by Budget Impact Analysis (BIA) to analyze different data sources and assess potential cost-savings from refining the EMHSLU.”

Revised: “*Taking WHO recommendations into consideration, we employed 1) a literature review to identify metrics used in peer-reviewed studies for measuring each criterion, and then to select context-appropriate metrics for Uganda, followed by 2) a Budget Impact Analysis (BIA) to analyze available data and assess potential cost-savings from refining the EMHSLU.*”

**2.3. Minor Areas of Improvement**

**Comment 1:** The background and motivation for the paper is rather weak. Please provide more detailed information on why this topic is important. For example, what is the process for assigning a health product to the EMHSL? What are the implications when a product is added to the list and when it is taken off? What is an essential medicine or health product? (Minor - I found the background and motivation for this paper to be quite strong as many LMIC governments would benefit from this more nuanced discussion of cost-effectiveness)

**Response:** We value the reviewer’s suggestion. We have added the following to the background and added references to each as well:

“Essential medicines are those that address the most critical healthcare needs of the population, considering factors such as disease prevalence, public health importance, evidence of effectiveness and safety, and cost-effectiveness.” - (ref #1)

“Changes to NEMLs can have several implications, including impacts on medicine availability, affordability, and prescribing practices” - (ref #10)

“The current process for assigning a health product to EMHSLU involves evaluation of the clinical efficacy, cost-effectiveness, public health relevance, and alignment with national guidelines by a national task force before final approval by the Ministry of Health.” - (ref #18)

**Comment 2:** Please provide some justification as why Uganda was selected as the case study. Why diabetes? This is valuable information that should not be left out. Further, how can your results be useful for other entities beyond the government of Uganda (e.g., other national governments, NGOs, etc.)? (Minor- I assume the investigators have ongoing collaborations in Uganda facilitating the availability of data for this analysis)

**Response:** Thank you for this helpful suggestion. We have already stated the rationale for selecting diabetes in the manuscript: *“T2DM is selected as a high-burden disease worldwide due to its high prevalence, mortality, and morbidity, and we focused on OHAs as they represent a significant portion of the DM management and control medication budget.”*

To address the reviewer’s request, we have revised the background to add a justification for selecting EMHSLU: *“We selected the Essential Medicines and Health Supplies List for Uganda (EMHSLU) as a well-structured NML, which provides a strong foundation assessment for our case study.”*

Lastly, we have revised the Discussion to highlight the broader applicability of our approach: *“The systematic approach used in this study to review the current medicines on the EMHSLU and develop recommendations—based on the WHO guideline for the selection of essential medicines at the country level—could serve as a useful model for other countries and disease areas.”*

**Comment 3:** In calculating the national supply availability, local vs. global production is a relevant factor that the paper has highlighted. Beyond that, I believe the proximity of global production supply base is another relevant factor that could be taken into consideration (this could determine the risk of potential disruptions). (minor)

**Response:** We thank the reviewer for drawing our attention to this issue. We have included the following sentence in the Limitations section and added references to support (ref #32 and ref#77):*“While our primary focus for availability was on the distinction between local and global production, other factors—such as global demand for certain medicines, the proximity of production sources, and regulatory compliance of manufacturers—may also influence the sustainability of supply chains for imported medicines.* *We also were unable to assess how changes in demand resulting from substitutions in the EMHSLU might influence medicine availability or accessibility in Uganda. Future studies should examine NEMLs that have associated demand data to further inform the optimization of NEMLs.”*

**Reviewer #2 Comments**

**Comment 1:** Distribution Challenges: The paper mentions that the inclusion of newer OHAs in the EMHSLU has led to issues of availability and accessibility, comparing them to older drugs like metformin or glibenclamide, which have a long history of use. While these challenges are significant, I believe it would be beneficial to emphasize the transitional nature of these issues. Strengthening and adapting the medication supply chain for newer drugs should be viewed as an expected part of the process, rather than framing it as a disadvantage of the new medications themselves. (moderate)

**Response:** We appreciate this insightful suggestion. We agree that the lower availability and accessibility of newer OHAs should be interpreted as a transitional challenge rather than an inherent disadvantage of the medicines themselves. We have added this point to the Discussion section under “Availability”: *“Although lower availability of medicines newly added to the EMHSLU is expected during the transition period,* *strengthening the functions of NRAs, such as registration and market authorization, licensing establishments, and market surveillance and control, will enhance the uninterrupted availability of medicines and ensure high-quality medicines are available in the country.*”

**Comment 2:** Glibenclamide Withdrawal: The MoH’s decision to remove glibenclamide from the essential medicines list, in line with current clinical practice guidelines due to safety concerns, is a key point. It would be beneficial to explore if the potential costs associated with treating complications like hypoglycemia, which could counterbalance the savings identified in the study. (Major- this is an important consideration that overlaps with the comments about the difference between gliclazide and other sulfonylureas)

**Response:** Thank you for highlighting this important consideration. We have added the following to our limitations with respect to the BIA: “*Lastly, this analysis did not account for the potential downstream costs associated with adverse clinical outcomes, such as hypoglycemia, which may be more common with certain medicines like glibenclamide and could impact some of the projected cost savings.*”

**Comment 3:** The authors describe that the availability of medications varies depending on the complexity of the hospital network. It would be interesting to explore how this is related to the clinical profiles of patients. Additionally, a more in-depth discussion on how clinical profiles and the clinical practice guidelines' recommendations could lead to greater efficiencies would be valuable. This could have an impact on the BIA calculations by better segmenting the patient population, ultimately generating higher cost savings. (Minor)

**Response:** We appreciate this thoughtful suggestion. To clarify how availability varies by level of care and may reflect differences in patient clinical profiles, we have added the following note: *“According to EMHSLU, each essential medicine is required to be accessible at certain levels of care, in line with the cascade of care. This requirement means that patients who require more advanced regimens for T2DM management are expected to receive these medicines at higher levels of care (i.e., secondary and tertiary health facilities). (See Table 3)”*

Additionally, we revised the Discussion section to better emphasize the role of clinical guidelines in driving efficiency and cost-effectiveness. The updated text reads: *“Aligning the national clinical guidelines with the NEML is necessary to ensure that the health workforce adheres to standardized treatment protocols and drug choices. This would enhance NEML efficiency and ultimately greater cost-savings by ensuring that patients receive appropriate medicines according to their clinical profiles.”* We have added a new reference to support this (ref #76)

**Comment 4:** Epidemiological Context: It would be helpful to introduce data on the burden of type 2 diabetes in Uganda earlier in the paper provide context for the study and its significance. (minor)

**Response:** Thank you for your suggestion. We have moved the following section from the conclusion on the burden of diabetes to the background: “*With a prevalence of 4.6% in 2021 and an estimated 48% of patients remaining undiagnosed, diabetes is considered a high-burden disease in Uganda*”

**Comment 5:** Repetition of Recommendations: Finally, the section "recommended changes to EMHSLU drug selections" repeats the recommendation to retain both doses of glimepiride and dapagliflozin (minor)

**Response:** Thank you for this careful observation. We have removed the repeated recommendation regarding glimepiride and dapagliflozin from the section on “Recommended changes to EMHSLU drug selections” to avoid redundancy.
