# Supplementary material for: Economic assessment of potential changes to essential medicines for diabetes in Uganda
Source: PLoS One. 2025 Jun 25;20(6):e0326806. doi: 10.1371/journal.pone.0326806 (PMC12193067; doi:10.1371/journal.pone.0326806)
Supplement: Attachment — Submitted filename: Response_to_Reviewers_auresp_2.docx [file pone.0326806.s006.docx]

Manuscript ID: PONE-D-25-01852R1
Title: *Economic Assessment of Potential Changes to Essential Medicines for Diabetes in Uganda*
Corresponding Author: Atousa Bonyani

We sincerely thank the Academic Editor and both reviewers for their detailed and constructive feedback on our manuscript. We greatly appreciate the insightful comments, which have contributed significantly to enhancing the clarity, accuracy, and overall quality of our work. Below, we address each point raised and have made the corresponding revisions in the manuscript using track changes.

**Reviewer #1 Comments**

**Comment 1:** On page 10, please correct the typo by changing the denominator from “tablet strenght” to “tablet strength.” (minor, please correct)

**Response:** We thank the reviewer’s comment. The typo is fixed.

**Comment 2:** Please number all equations accordingly. (minor, please correct)

**Response:** Thank you for this valid comment. We have added the equation numbers to the manuscript.

**Comment 3:** In Table 8, the recommended change for “Glibenclamide 5 mg” is to “Keep” the drug. However, this drug was already taken off the essential medicine list in 2023. Would it not make more sense if the recommendation is to instead “Add” this drug? (minor, please explain)

**Response:** We appreciate this important point. Since the medicine is still in the health system and the government is in the transition phase to remove glibenclamide, we used the word “Keep” instead of “Add”.

**Comment 4:** Would it be possible to create a table similar to table 8 but with summary information on the (i) justifications for the recommended changes (e.g., cost savings, lack of clinical benefits, etc.) along with the (ii) pragmatic and managerial challenges for implementing these recommendations (e.g., lack of local production capacity, etc.)? That would provide the reader with a concise overarching picture of all they need to know regarding the paper’s actionable implications. (minor, please consider as this would greatly improve clarity)

**Response:** We truly appreciate the reviewer’s thoughtful comment. We have added a new column to Table 8 titled “Justification for the Recommended Changes.” Regarding the pragmatic and managerial challenges, we believe that presenting this information in the text is more helpful to readers, as a table may not add additional clarity.
